# 

**Published:** 1890-05-03

**Authors:** 


SATURDAY, MAY 3rd, 1890.

**Epsom College** - - - - - - - - - - 59

**Words of Consolation.**—Patience - - - - - - 60

**Education and Benevolence for Doctors.** By Sir James Paget, Bart., F.R.S. - - - - - - - - 61

**The History and Capabilities of Herbal Simples.** By W. T. Fernie, M.D. VIII.—Blackberries—Brooklime - 62

**The Doctor's Pulpit.**—A Dead Journalist - - - - - 63

**May** - - - - - - - - - - - - - 64

**Everybody's Page** - - - - - - - - - - 65

**Notes and News** - - - - - - - - - - 66

**The Sussex County Hospital** - - - - - - - 67

**Passing Topics.**—The New Hospital for Women - - - 72

**Annotations.**—Dégraissage—Hop sand their Rivals—Women's Chronic Diseases - - - - - - - - - - 68

**The Practitioner's Mirror and Retrospect:**

Medicine—Solution of Gall Stones—Salicylates in Pleurisy—Paralysis after Diphtheria—Poisoning by Bad Meat - 69

Surgery — Aspiration in Pneumothorax — Pneumotomy—Phosphorus Necrosis — Splenectomy — Pulsating Exophthalmos—Inflammatory Exophthalmos—A Rare Case of Symblepharon—Glaucoma in Early Life - - - - - 69

Therapeutics—Sodium *versus* Potassium Salts - - - - 71

**Scraps and Gleanings** - - - - - - - - - 72

**The Student's Column.**—Durham University - - - - 72

EXTRA SUPPLEMENT—THE NURSING MIRROR.

**En Passant.**—British Nurses' Association—Uniforms for June—Nurses' Missionary Association—Staffordshire Institution—American Doctors and Nurses—Richmond Institution—American Papers—Short Items—A Suggestion for the Cape Government - - - - - - xvii
**Nursing Lectures.** By Percy Lewis, M.D. X.—The Skin - - - - - - - - - - - - xviii
**Death in Our Ranks** - - - - - - - - - xviii
**The Passing of the Shadow** (*concluded*) - - - - - xix
**Everybody's Opinion.**—The Junius S. Morgan Memorial—Certificates, Not Policies - - - - - - - - xix
**Presentations.—Notes and Queries** - - - - - xx
**Vacancies.—Appointments** - - - - - - - xx
**Amusements and Relaxation** - - - - - - - xx

